# Maternal high-fat diet decreases milk endocannabinoids with sex-specific changes in the cannabinoid and dopamine signaling and food preference in rat offspring

**DOI:** 10.3389/fendo.2023.1087999

**Published:** 2023-02-28

**Authors:** Camilla P. Dias-Rocha, Julia C. B. Costa, Yamara S. Oliveira, Larissa B. Fassarella, Juliana Woyames, Georgia C. Atella, Gustavo R. C. Santos, Henrique M. G. Pereira, Carmen C. Pazos-Moura, Mariana M. Almeida, Isis H. Trevenzoli

**Affiliations:** ^1^ Laboratório de Endocrinologia Molecular, Instituto de Biofísica Carlos Chagas Filho, Universidade Federal do Rio de Janeiro, Rio de Janeiro, Brazil; ^2^ Laboratório de Bioquímica de Lipídios e Lipoproteínas, Instituto de Bioquímica Médica Leopoldo de Meis, Universidade Federal do Rio de Janeiro, Rio de Janeiro, Brazil; ^3^ Laboratório de Desenvolvimento Tecnológico, Instituto de Química, Universidade Federal do Rio de Janeiro, Rio de Janeiro, Brazil

**Keywords:** maternal high-fat diet, metabolic programming, breast milk, endocannabinoid system, obesity, rat development, food preference, dopamine system

## Abstract

**Introduction:**

Maternal high-fat (HF) diet during gestation and lactation programs obesity in rat offspring associated with sex-dependent and tissue-specific changes of the endocannabinoid system (ECS). The ECS activation induces food intake and preference for fat as well as lipogenesis. We hypothesized that maternal HF diet would increase the lipid endocannabinoid levels in breast milk programming cannabinoid and dopamine signaling and food preference in rat offspring.

**Methods:**

Female Wistar rats were assigned into two experimental groups: control group (C), which received a standard diet (10% fat), or HF group, which received a high-fat diet (29% fat) for 8 weeks before mating and during gestation and lactation. Milk samples were collected to measure endocannabinoids and fatty acids by mass spectrometry. Cannabinoid and dopamine signaling were evaluated in the nucleus accumbens (NAc) of male and female weanling offspring. C and HF offspring received C diet after weaning and food preference was assessed in adolescence.

**Results:**

Maternal HF diet reduced the milk content of anandamide (AEA) (*p<0.05*) and 2-arachidonoylglycerol (2-AG) (*p<0.05*). In parallel, maternal HF diet increased adiposity in male (*p<0.05*) and female offspring (*p<0.05*) at weaning. Maternal HF diet increased cannabinoid and dopamine signaling in the NAc only in male offspring (*p<0.05*), which was associated with higher preference for fat in adolescence (*p<0.05*).

**Conclusion:**

Contrary to our hypothesis, maternal HF diet reduced AEA and 2-AG in breast milk. We speculate that decreased endocannabinoid exposure during lactation may induce sex-dependent adaptive changes of the cannabinoid-dopamine crosstalk signaling in the developing NAc, contributing to alterations in neurodevelopment and programming of preference for fat in adolescent male offspring.

## 1 Introduction

Nutritional, hormonal, or environmental adversities during critical periods of development, such as gestation and lactation, increase the risk for chronic diseases across the lifespan (1, 2). This phenomenon is known as metabolic programming and has been implicated in the developmental origins of obesity, diabetes, and hypertension in humans and experimental models (3, 4).

Obesity is associated with an overactivation of the endocannabinoid system (ECS), which is also a hallmark of neurodevelopment (5–7). The major lipid endocannabinoids are anandamide (AEA) and 2-arachidonoylglycerol (2-AG) that bind to cannabinoid receptor type 1 (CB1) and type 2 (CB2). AEA and 2-AG are synthesized “on demand” by the enzymes N-acylphosphatidylethanolamine (NAPE)-phospholipase D hydrolase (NAPE-PLD) and diacylglycerol lipase (DAGL), respectively, from membrane phospholipids containing arachidonic acid. AEA is preferentially degraded by the fatty acid amide hydrolase (FAAH) while 2-AG is mostly metabolized by monoacylglycerol lipase (MAGL). Along with these major components, others bioactive lipids as oleoylethanolamide (OEA) and palmitoylethanolamide (PEA), and a complex network of receptors comprise the “paracannabinoid system” or the “endocannabinoidome” (6, 8).

In the developing brain, the ECS regulates neurogenesis, cell lineage commitment, neuronal migration, axonal elongation, synaptogenesis, glial formation, and postnatal myelination (7, 9). The ECS is expressed in the human brain as early as gestational week nine (10) and in rodent brain from gestational day twelve (11), evidencing the importance of the ECS regulation during gestation and lactation. Early adversities in nutrition (over or undernutrition) or exposure to environmental toxicants (tobacco, cannabis or alcohol) can modulate the ECS components in the brain and peripheral tissues throughout life, in a sex-specific manner, programming energy metabolism and behavior as compiled in a recent review (7).

The ECS stimulates food intake, the appetite for fat (12, 13) and increases adiposity (14, 15). Food intake regulation also has a major contribution of several peripheral hormones such as the adipose-derived factor leptin, insulin, and gut hormones as peptide YY (PYY) and glucagon-like peptide 1 (GLP-1), all presenting anorexigenic effect (13, 16). The hypothalamus is the principal brain region involved in the homeostatic regulation of food intake while mesolimbic regions (nucleus accumbens, NAc; ventral tegmental area, VTA) are important modulators of the hedonic eating and motivational behaviors, with participation of the cannabinoid and dopamine signaling crosstalk (13, 17, 18).

In rats, there is an important maturation of the feeding circuitry during the early postnatal period and leptin (serum level peaks around postnatal day 10) has a marked role in the developing hypothalamus with sex-divergent responses (19–21). Leptin is present in the breast milk of humans (22) and rodents (23, 24). Despite the fluctuations in the serum levels of rodent neonates, the breast milk leptin levels remain relatively constant across lactation (24), suggesting that serum leptin peak is more influenced by the offspring adipose production rather than milk ingestion.

It has been suggested that other milk components (nutrients and bioactive molecules) during neonatal period could modulate brain maturation (25–27). The lipid endocannabinoids AEA and 2-AG and other endocannabinoid-like lipids have also been identified in human milk (28–30) but their physiological role remains to be elucidated. On the other hand, in rodents, it has been demonstrated that cannabinoid signaling is important for suckling initiation (31) but the presence of these lipids in rat breast milk had not been demonstrated.

We have previously shown that maternal high-fat (HF) diet programs early obesity in rat offspring (23), with sex-dependent changes of the ECS in the hypothalamus (32, 33), white and brown adipose tissue (32, 34, 35), and liver (36). In the present study, we hypothesized that maternal HF diet would increase the lipid endocannabinoid levels in breast milk programming cannabinoid and dopamine signaling in the NAc of weanling rats and food preference in adolescence, which is a critical window for both development and ECS modulation (9). To test our hypothesis, we used a well characterized rat model of metabolic programming induced by maternal intake of HF diet from preconception to lactation.

## 2 Materials and methods

### 2.1 Animal model and diets

Twenty 60-day-old female Wistar rats weighing 180-220 g (female progenitors) and ten 100-day-old male Wistar rats weighing 250-300 g (male progenitors) were obtained from the Center of Reproduction Biology of the Federal University of Rio de Janeiro, Rio de Janeiro, Brazil. All animal procedures met National Institutes of Health guide for the care and use of Laboratory animals (NIH Publications No. 8023, revised 1978) and were approved by the Animal Care and Use Committee of the Health Science Center of the Federal University of Rio de Janeiro (process number 059/19). For all animal procedures, rats were kept in a controlled temperature environment (23 ± 2 °C) with a photoperiod of 12 hours (7 a.m. to 7 p.m. – light, and 7 p.m. to 7 a.m. - dark). Water and the experimental diets were offered *ad libitum* throughout the study.

Progenitor female rats were randomly assigned to two dietary treatments (n=10/group): Control group (C), which received a standard diet for rodents (10.9% of the calories as fat), and a high-fat group (HF), which received a high-fat diet (28.7% of the calories as fat) (**Table 1**). In the HF diet, lard was used as fat source, and we also added soy oil to provide the minimal amount of polyunsaturated fatty acids for adequate development of rats. We have recently published that the HF diet contains higher saturated fatty acid than control diet (+2.7-fold, *p<0.05*), including more C12:0 (+15.3-fold, *p<0.05*), C14:0 (+13.9-fold, *p<0.05*), C16:0 (+86.7%, *p<0.05*) and C18:0 (+5.7-fold, *p<0.05*) (37). C and HF diets contain 3.34 kcal/g and 3.65 kcal/g, respectively (**Table 1**). Both diets followed the AIN-93G recommendations (23, 32–34, 38, 39). Female rats were fed these diets during 8 weeks before mating (2 females to 1 male), and throughout gestation and lactation. This experimental design aimed to isolate the effect of maternal dietary insult (HF diet) during early development (gestation and lactation) on the offspring metabolic outcomes and ECS regulation in the NAc, an important brain area for food intake and preference.

**Table 1:** Composition of the control (C) diet and high-fat (HF) diet.

	C diet - Nuvilab^®^		
	(g)	Kcal	% Kcal
Protein	220	880	26.31
Lipid	40	364	10.89
Carbohydrate	525	2100	62.80
Mineral	90	0	0
Humidity	125	0	0
Energy	**-**	**3.34 kcal/g**	**-**
	HF diet		
	**(g)**	**Kcal**	**% Kcal**
C diet - Nuvilab	150	501	**-**
Condensed milk	395	1267.99	**-**
Skim powdered milk	280	952	**-**
Maize starch	150	571.5	**-**
Soy oil	9.3	83	**-**
Lard	80	720	**-**
AIN-93G Mix Mineral^1^	29	0	**-**
AIN-93G Mix Vitamin^1^	8	0	**-**
Choline^2^	0.82	0	**-**
L-Cystine^2^	2.625	0	**-**
BHT^2^	0.1	0	**-**
Total	1121.5	4096.79	**-**
	**(g)**	**Kcal**	**% Kcal**
Protein	155.6	622.4	15.19
Lipid	130.8	1177.2	28.73
Carbohydrate	582.1	2328.4	56.83
Energy	**-**	**3.65 kcal/g**	**-**

^1^Rhoster (Araçoiaba da Serra, SP, Brazil) and ^2^Farmos (Rio de Janeiro, RJ, Brazil). BHT, Butylated Hydroxytoluene. Bold values highlight the total energy contents of the diets in kcal per gram of diet.

Pregnant rats were housed in individual standard rat cages. At birth, all the litters were adjusted to three males and three females per dam to standardize and adequate the milk supply/demand among the litters (40, 41). The remaining neonate pups from each litter were used for serum hormonal analysis. At weaning, a subset of male and female offspring was euthanized for adiposity evaluation by weighting the retroperitoneal fat pad as representative of visceral adipose tissue (VIS) and the inguinal fat pad as representative of subcutaneous adipose tissue (SUB). Serum hormonal profile and the ECS and dopamine signaling in the Nac of weanling male and female offspring were analyzed.

Dams were weighted before mating and during pregnancy and lactation weekly. Milk samples were collected at the postnatal day 11 (mid-lactation) and 21 (late lactation) under oxytocin (5 IU/mL) and anesthesia (55 mg/Kg of ketamine and 100 mg/Kg of xylazine) (Aché, Brazil). For milk extraction, lactating rats were separated from their offspring for 2 hours to maximize milk volume. The milk samples were extracted by gently squeezing the thoracic and abdominal teats and were stored at -80°C, as we previously described (23). Milk samples were used for quantification of fatty acids and lipid endocannabinoids by mass spectrometry. At weaning, lactating rats were also euthanized, and serum and mammary gland samples were collected.

**Figure 1:**
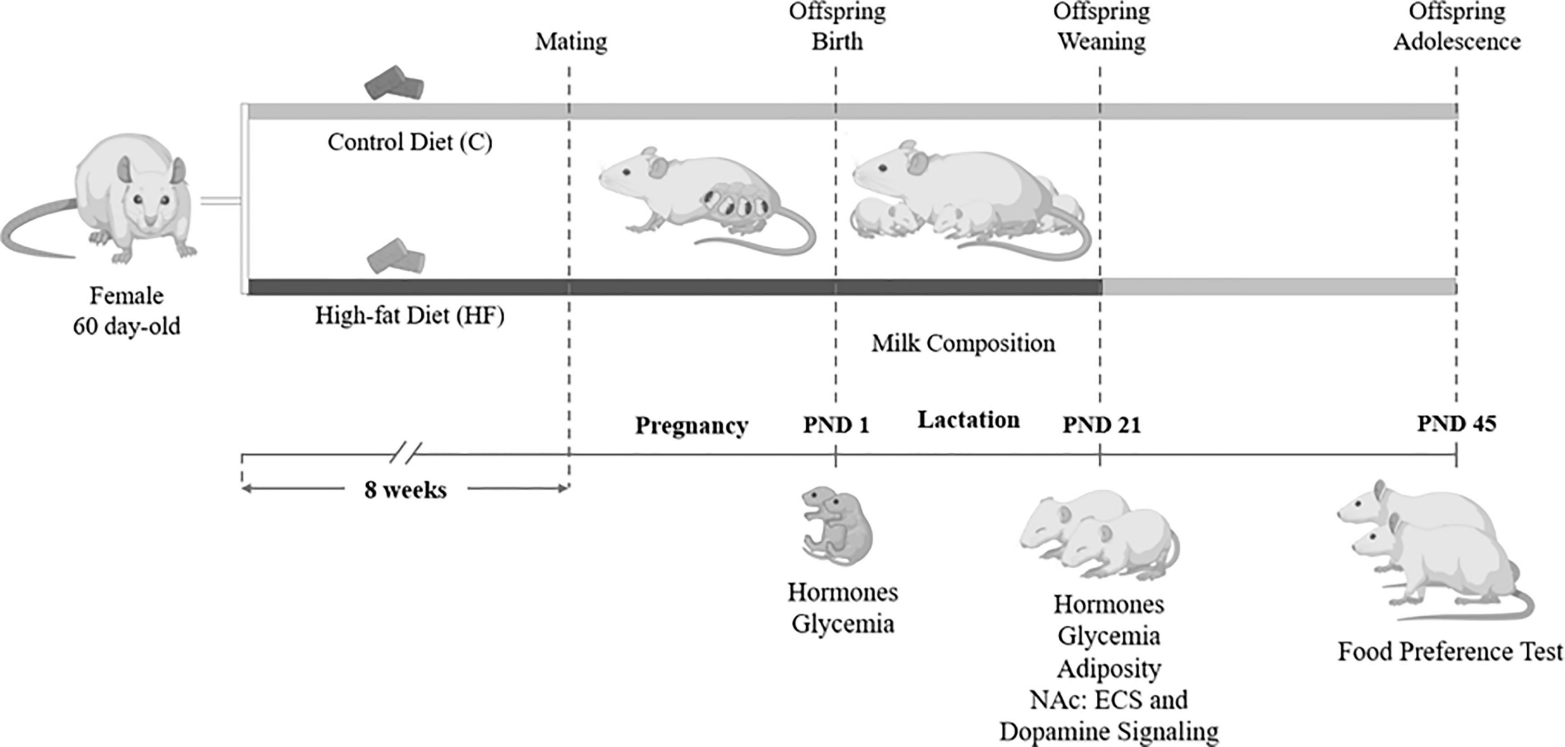
Experimental model of maternal high-fat diet and study design. Female Wistar rats (60 day-old) were divided into control group (C, control diet) or High-fat group (HF, high-fat diet). Dams received C or HF diet for 8 weeks prior mating and throughout gestation (21 days) and lactation (21 days). At birth (postnatal day 1, PND 1), male and female pups underwent euthanasia for hormone and glycemia measurement. During lactation, milk samples were collected for endocannabinoid and fatty acid quantification. At weaning (PND 21), a subset of male and female offspring was killed for hormone, glycemia and adiposity evaluation, and analysis of the endocannabinoid system (ECS) and dopamine signaling in the Nucleus Accumbens (NAc). In the adolescence (PND 45), another subset of the offspring was submitted to a food preference test.

From weaning (postnatal day 21) until adolescence (postnatal day 45), both C and HF offspring received control diet to isolate the effect of maternal dietary insult (HF diet) during early development (gestation and lactation) on the offspring metabolic outcomes and ECS regulation. Offspring were weighted every three days until weaning and weekly after weaning. In the postnatal day 45, male and female offspring were tested for food preference as previously described (32). Briefly, rats were allowed to access control, HF or high-sugar (HS; 30% sucrose) diets simultaneously for 24h. Food intake was recorded to evaluate preference for dietary fat or sugar. The timeline of the animal procedures is described in **Figure 1**.

Euthanasia of all animals occurred between 9 a.m. and 12 p.m. in a fed state by decapitation and serum obtained from trunk blood. The glycemia was measured using a glucometer (Accu-Chek, Roche, Switzerland). Serum samples were stored at −80°C. For each experimental procedure, rats from at least five different litters per group were used to avoid “litter effects” on the statistical analysis.

### 2.2 Serum lipid and hormonal profile

Blood samples were collected and centrifuged (1233×g for 15 min, 4°C) for serum separation. Total triglycerides and cholesterol were measured by an enzymatic colorimetric kit (Bioclin, Quibasa Química, MG, Brazil). Leptin, insulin, PYY and GLP-1 levels were determined using a specific rat milliplex Kit from Merck Millipore (cat# RMHMAG-84K, MA, USA), with assay sensitivity of 8 pg/mL for leptin, 14 pg/mL for insulin, 1 pg/mL for PYY and 28 pg/mL for GLP-1. Intra-assay coefficient of variation was < 10% for all analytes. We evaluated 5-7 samples per group.

### 2.3 Milk endocannabinoid and fatty acid quantification

AEA and 2-AG milk concentration was assessed by liquid chromatography–mass spectrometry based on the method previously described for human milk (28) with slight adaptations. The assay was conducted using 150μL of whole rat milk or 150μL of each standard of the calibration curve. The standard curve was obtained by serial dilution of the AEA and 2-AG standards (Cayman Chemical, *USA*) in a 10% milk powder solution in the range of 0.03-5.0 ng/mL for AEA and 23-3,000 ng/mL for 2-AG. The deuterated standards AEAd4 (1 ng) and 2-AGd5 (10 ng) (Cayman Chemical, *USA*) were added to each sample or standards as internal calibrators (20μL). An ice-cold mixture of acetonitrile and PBS (1:1) was added to the samples and standards (1,000 μL) for protein precipitation and lipid extraction. The proteins were separated by centrifugation (14,000 x g, 4°C, 5 minutes). The supernatant was acidified with 5 volumes of 5% phosphoric acid. A solid phase extraction was performed using reversed phase chromatography cartridges OASIS HLB (Waters Corp., Mildford, MA, USA) following manufacturer’s instructions. The lipid fraction was eluted from the columns with 1 mL of acetonitrile at room temperature. The eluate was dried under nitrogen steam at 32°C for 20 min and resuspended in 100μL of methanol for the endocannabinoid detection (in triplicate) by LC-HRMS.

The chromatographic separation was performed in a reversed-phase column ACE CORE-25A-0502U (2.5μm, 50mm x 2.1mm) at 40°C. The mobile phases were composed of (A) H_2_O with 5 mM ammonium formate and 0.1% formic acid, (B) MeOH with 0.1% formic acid. The flow rate was set at 500 µL.min^-1^. The elution profile was 0-0.5min, 75% B; 0.5-2 min, 75-100% B; 2-4 min, 100% B; 4-4.1min, 75% B; 4.1-5.0, 75% B to equilibration to the initial conditions. The overall run time was 5 minutes, and the injection volume was 20.0 µL. The LC effluent was pumped to a Q-Exactive mass (Q Exactive Plus - Ultimate 3000 HPLC system, Thermo spectrometer Scientific Germany) operating in positive ionization mode. The spray voltage was set at 3.9 kV and 2.9 kV in positive ionization mode. The capillary temperature was 380°C, and the S-lens radio frequency (RF) level was set at 80 (arbitrary units). The nitrogen sheath and auxiliary gas flow rates were set at 60 and 20 (arbitrary units), respectively. To ensure mass accuracies below 6 ppm, the instrument was calibrated in positive and negative mode using the manufacture’s calibration solutions (Thermo Fisher Scientific, Bremen, Germany). The mass spectrometer acquired FullMS and T-SIM at resolution of 70,000 full width at half maximum (FWHM) and with an automatic gain control (AGC) of 106, maximum IT 75ms. The target mass was 348.2890 m/z to AEA, 379.2837 m/z to 2-AG, 352.3143 m/z to D4-AEA and 384.3153 m/z to D5-2AG. For this analysis, we used 5-8 samples per group.

Fatty acid profile in milk samples was assessed by gas chromatography-mass spectrometry based on the method previously described (33, 42). The lipid samples were homogenized in a toluene and 1% sulfuric acid in methanol solution. GC-MS analysis was carried out on a Shimadzu GCMS-QP2010 Plus system, using an Agilent column (25 m x 0.20 mm x 0.33 μm), HP Ultra 2 (5% Phenyl-methylpolysiloxane). Injector was set at 250°C and column temperature was programmed from 40–160°C at 30°C/min, 160-233°C at 1°C/min, 233-300°C at 30°C/min and held at 300°C for 10 min. Electro ionization (EI-70 eV) and a quadruple mass analyzer, operated in scans from 40 to 440 amu. Interface was set at 240°C and the ion source at 240°C. The components were identified by comparing their mass spectra with those of the library NIST05 contained in the computer’s mass spectrometer. Retention indices were also used to confirm the identity of the peaks in the chromatogram by Supelco 37 Component FAME Mix (Sigma-Aldrich). Fatty acids were quantified by determining peak-area ratios with the 9:0 and 19:0 internal standards. We used 3-5 samples per group.

### 2.4 Microdissection of the nucleus accumbens

The whole brain of weanling rats was carefully removed and conditioned to -20°C for subsequent dissection in cryostat by the punch technique as we previously described for hypothalamic nuclei (23, 43), with adaptations for the NAc. The NAc punch samples were obtained from thick coronal brain sections using the bregma as reference (44) and the rat brain atlas (45). Briefly, four subsequent sections of 500 μM were made: from Bregma +2.76 mm to Bregma +0.72 mm for microdissection of two subregions of the NAc, i.e., core (cNAc) and shell (sNAc). The NAc was dissected bilaterally from all sections in one punch with a 1.5 mm-diameter round needle using the lateral ventricles and 1.5 mm from the base of the brain as references. Immediately afterwards, the tissue samples were kept at −80°C until Western blotting assays.

### 2.5 Western blotting

Western blotting was used to investigate the protein content of the ECS components in the mammary gland from dams and in the NAc of offspring (32–34). In addition, the dopamine signaling was also evaluated in the NAc of weanling offspring. The protein content of glyceraldehyde-3-phosphate dehydrogenase (GAPDH) or cyclophilin was used as loading control.

The mammary gland samples were homogenized in pH 7.4 lysis buffer (20mM TRIS-HCl, 10mM NaF, 1% NP40, 150mM NaCl, 5mM EDTA, 0.1% SDS) and the NAc samples were homogenized in pH 7.4 lysis buffer (50 mM Tris, 150 mM NaCl, 1% Triton 100×, 0.1% SDS, 5 mM EDTA, 50 mM NaF, 30 mM sodium pyrophosphate, 1 mM sodium orthovanadate) both containing protease inhibitor cocktail (Thermo Scientific, catalog number A32959). After centrifugation, the total protein content of supernatant was quantified using the PierceTM BCA Protein Assay Kit (Thermo Scientific, Rockford, USA).

The samples were denatured in sample buffer (50mM Tris-HCl, pH 6.8, 1% SDS, 5% 2-mercaptoethanol, 10% glycerol, 0.001% bromophenol blue) and heated at 95°C for 5 min. Total proteins were analyzed by SDS-PAGE, with a 12% polyacrylamide gel, and transferred onto polyvinylidene difluoride membranes (Hybond-P 0.45 μm PVDF; Amersham Biosciences BKM, ENG). The membranes were incubated with T-TBS containing 5% Bovine Serum Albumin (Sigma Life Science MO, USA) for 90 minutes to block non-specific binding sites. Then, the membranes were incubated overnight at 4°C with specific primary antibodies. Membranes were washed and incubated for 2 hours at room temperature with peroxidase labeled specific secondary antibodies. All blots were washed and incubated with a luminogen detection reagent (Amersham ECL Prime Western Blotting Detection reagent; Amersham Bioscience, Inc). Information about primary and secondary antibodies is described in **Table 2**. Chemiluminescent signal was detected by ImageQuant LAS 4000 equipment followed by densitometric analyses (GE Healthcare Life Sciences). Data are expressed as percentage of control male group (set at 100%). For each protein analyzed by Western blotting, we evaluated 6 or 7 samples per group.

**Table 2:** Primary and secondary antibodies used for Western Blot.

Primary Antibodies			Secondary Antibodies		
	Company	Dilution	Company	Dilution	Specificity
**CB1** *Cat #101500*	Cayman MI, USA	1:500	Amersham Bioscience *Cat #NA934*	1:5000	Anti-rabbit
**CB2** *WH0001269M1*	Sigma MO, USA	1:1000	Cell Signaling MA, USA *Cat #7076*	1:10000	Anti-mouse
**FAAH** *Cat #101600*	Cayman MI, USA	1:200	Amersham Bioscience *Cat # NA934*	1:5000	Anti-rabbit
**MAGL** *Cat #sc-398942*	Santa Cruz CA, USA	1:1000	Amersham Bioscience *Cat # NA934*	1:10000	Anti-rabbit
**NAPE-PLD** *Cat #10305*	Cayman MI, USA	1:200	Invitrogen CA, USA *Cat #31460*	1:5000	Anti-rabbit
**DAGLα** *Cat #sc-390409*	Santa Cruz CA, USA	1:100	Cell Signaling MA, USA *Cat #7076*	1:10000	Anti-mouse
**D1** *Cat #NBP2-162113*	Novus Biologicals CO, USA	1:500	Invitrogen CA, USA *Cat #31460*	1:5000	Anti-rabbit
**D2** *Cat #NB600-1261*	Novus Biologicals CO, USA	1:500	Invitrogen CA, USA *Cat #31460*	1:5000	Anti-rabbit
**DAT** *Cat #ab184451*	Abcam Cambridge, UK	1:1000	Invitrogen CA, USA *Cat #31460*	1:5000	Anti-rabbit
**TH** *Cat #AB152*	Merck Millipore MA, USA	1:1000	Amersham Bioscience *Cat # NA934*	1:10000	Anti-rabbit
**DARPP-32** *Cat #AB10518*	Merck Millipore MA, USA	1:1000	Amersham Bioscience *Cat # NA934*	1:5000	Anti-rabbit
**β-Actin** *Cat #sc-1615*	Santa Cruz CA, USA	1:5000	Invitrogen CA, USA *Cat # 31402*	1:10000	Anti-goat
**GAPDH** *Cat #2118*	Cell Signaling MA, USA	1:5000	Amersham Bioscience *Cat #NA934*	1:10000	Anti-rabbit
**Cyclophilin** *Cat #PA1-027A*	ThermoFisher MA, USA	1:5000	Amersham Bioscience *Cat #NA934*	1:10000	Anti-rabbit

CB1, cannabinoid type-1 receptor; CB2, cannabinoid type-2 receptor; FAAH, fatty acid amide hydrolase; MAGL, monoacylglycerol lipase; DAGLα, diacylglycerol lipase α; NAPE-PLD, n-acyl-phosphatidylethanolamine-hydrolyzing phospholipase D; D1, dopamine type-1 receptor; D2, dopamine type-2 receptor; DAT, dopamine transporter; TH, tyrosine hydroxylase; DARPP-32, Dopamine- and cAMP-regulated neuronal phosphoprotein; GAPDH, glyceraldehyde-3-phosphate dehydrogenase.

### 2.6 Statistical analysis

The statistical analysis was performed using the software GraphPad Prism 8 (GraphPad Software Inc., CA, USA). For all analyses, normality was assessed by the Kolmogorov-Smirnov test and Grubb’s test was used to detect outliers.

The unpaired Student’s *t* test was used for comparisons between C and HF dams and to analyze the food preference in adolescent offspring. Two-way ANOVA with Bonferroni’s *post hoc* test was used to analyze offspring data, considering maternal diet or offspring sex as main factors. Statistically significant differences were considered when *p<0.05*. Results are shown as mean ± standard deviation. For the western blotting assays data are expressed as percentage of change compared to control dam or male group set as 100%.

## 3 Results

### 3.1 Maternal metabolic phenotype

**Figure 2:**
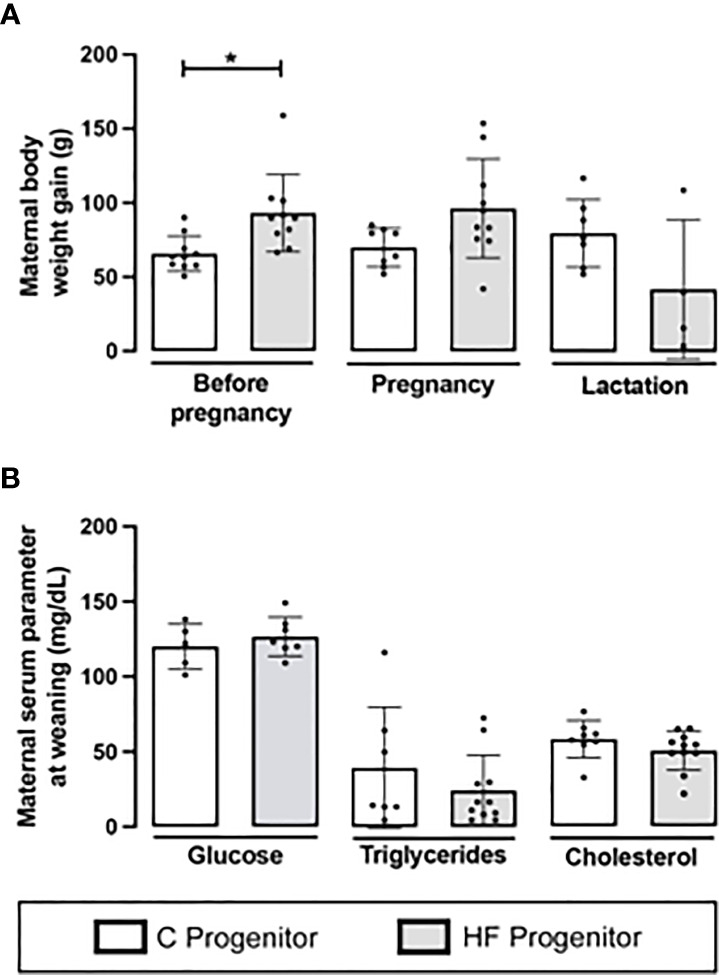
Effect of high-fat (HF) diet on the body weight and serum metabolites of rat female progenitors. **(A)** Maternal body weight gain for 8 weeks prior conception, and during pregnancy and lactation; and **(B)** Serum glucose, triglycerides, and cholesterol levels of control (C) and HF dams at weaning. Data are presented as mean ± standard deviation and statistically significant differences were determined by unpaired Student’s *t* test. *p < 0.05.

Before gestational period, C and HF dams received dietary treatments for 8 weeks and the HF diet increased the body weight gain compared to C diet (+41%, *p<0.05*). However, there were no differences in the maternal body weight gain during pregnancy and lactation (**Figure 2A**). At weaning, HF diet did not alter maternal serum levels of glucose, triglycerides, or cholesterol (**Figure 2B**).

### 3.2 Offspring metabolic phenotype

**Figure 3:**
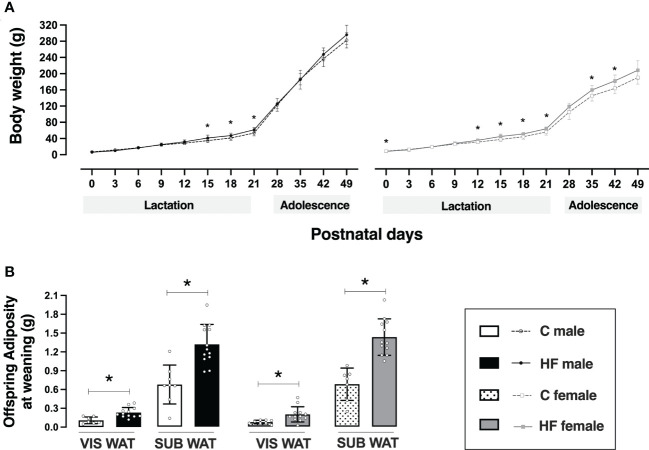
Effect of maternal high-fat (HF) diet on offspring body weight and adiposity in the early life. **(A)** Body weight and **(B)** visceral (VIS) and subcutaneous (SUB) adiposity of control (C) and HF male and female offspring at weaning (postnatal day 21). Data are presented as mean ± standard deviation and statistically significant differences were determined by two-way ANOVA followed by Bonferroni test. *p < 0.05.

Maternal HF diet slightly decreased birth weight in female offspring (-8%, *p<0.05*) but did not alter birth weight of male pups (**Figure 3A**). During lactation, maternal HF diet increased body weight of male offspring at postnatal day 15 (+19%, *p<0.05*), 18 (+15%, *p<0.05*) and 21 (+15%, *p<0.05*), compared to C offspring (**Figure 3A**). In the female offspring, maternal HF diet induced a “catch up” of the body weight during lactation, with increased body weight of female HF offspring at postnatal day 12 (+14%, *p<0.05*), 15 (+20%, *p<0.05*), 18 (+16%, *p<0.05*) and 21 (+14%, *p<0.05*), compared to C offspring (**Figure 3A**).

Maternal HF diet did not change body weight of male offspring from weaning to adolescence, but female HF offspring remained with increased body weight compared to their sex-matched controls at postnatal day 35 (+10%, *p<0.05*) and 42 (+11%, *p<0.05*) (**Figure 3A**).

Maternal HF diet increased VIS WAT mass (+ 2.1-fold, *p<0.05*) and SUB WAT mass (+ 95%, *p<0.05*) of male offspring at weaning compared to C offspring (**Figure 3B**). A similar profile was observed in female HF offspring, with increased VIS WAT mass (+ 2.7-fold, *p<0.05*) and SUB WAT mass (+ 2.1-fold, *p<0.05*), compared to C offspring (**Figure 3B**).

Maternal HF diet did not change the glycemia or hormonal profile of newborn male or female offspring (**Table 3**). However, maternal HF diet increased the glycemia and insulinemia of weanling offspring (maternal diet effect *p<0.05*). The *post hoc* analysis of glycemia showed a statistically increase in male (+ 24%, *p<0.05*) and female (+ 14%, *p<0.05*) offspring compared to their sex-matched controls at weaning. Maternal HF increased the serum levels of insulin in the offspring (maternal diet effect *p<0.05*), with statistically difference in the *post hoc* test for males (+ 3.6-fold, *p< 0.05*) compared to sex-matched controls, but maternal HF diet did not change the serum levels of leptin, PYY or GLP-1 (**Table 3**).

**Table 3:** Effect of maternal high-fat diet (HF) on serum metabolites of male and female offspring at birth and weaning.

		Serum metabolites (Mean ± SD)				Source of variation		
		C male	HF male	C female	HF female	MD	S	MD x S
**Offspring at birth**	Glycemia (mg/dL)	59.0 ± 12.3	60.2 ± 11.2	57.6 ± 12.7	62.6 ± 11.8	0.34	0.87	0.55
	Leptin (ng/mL)	1.27 ± 0.73	0.76 ± 0.50	0.92 ± 0.61	0.99 ± 0.84	0.48	0.83	0.34
	Insulin (ng/mL)	0.75 ± 0.39	1.05 ± 0.53	1.09 ± 0.44	1.27 ± 0.71	0.32	0.25	0.79
	PYY (ng/mL)	4.75 ± 4.36	3.33 ± 2.46	4.10 ± 4.51	4.19 ± 4.17	0.70	0.95	0.66
	GLP-1 (ng/mL)	0.88 ± 0.70	1.43 ± 1.36	1.38 ± 0.94	1.79 ± 1.78	0.39	0.44	0.89
**Offspring at weaning**	Glycemia (mg/dL)	114 ± 15.0	141 ± 16.4*	119 ± 12.5	136 ± 15.9*	*<0.05*	0.94	0.14
	Leptin (ng/mL)	2.83 ± 0.57	3.10 ± 1.00	2.46 ± 0.49	2.70 ± 0.70	0.37	0.19	0.97
	Insulin (ng/mL)	0.81 ± 0.43	2.90 ± 1.06*	1.30 ± 0.73	2.20 ± 0.92	*<0.05*	0.74	0.08
	PYY (pg/mL)	94.0 ± 14.0	153 ± 93.2	84.4 ± 9.65	90.0 ± 36.9	0.15	0.11	0.23
	GLP-1 (ng/mL)	0.15 ± 0.03	0.14 ± 0.03	0.13 ± 0.04	0.13 ± 0.03	0.66	0.31	0.50

Data presented as mean ± standard deviation. Statistically significant differences were determined by two-way ANOVA followed by Bonferroni’s *post hoc* test, considering *p < 0.05*. Differences between control (C) and high-fat (HF) groups in the *post hoc* test are presented as *p < 0.05. n=5-7 samples per group. PYY, Peptide YY; GLP-1, Glucagon-like peptide-1, MD, Maternal diet, S, Offspring sex.

### 3.3 Milk composition and the ECS in the mammary gland

**Figure 4:**
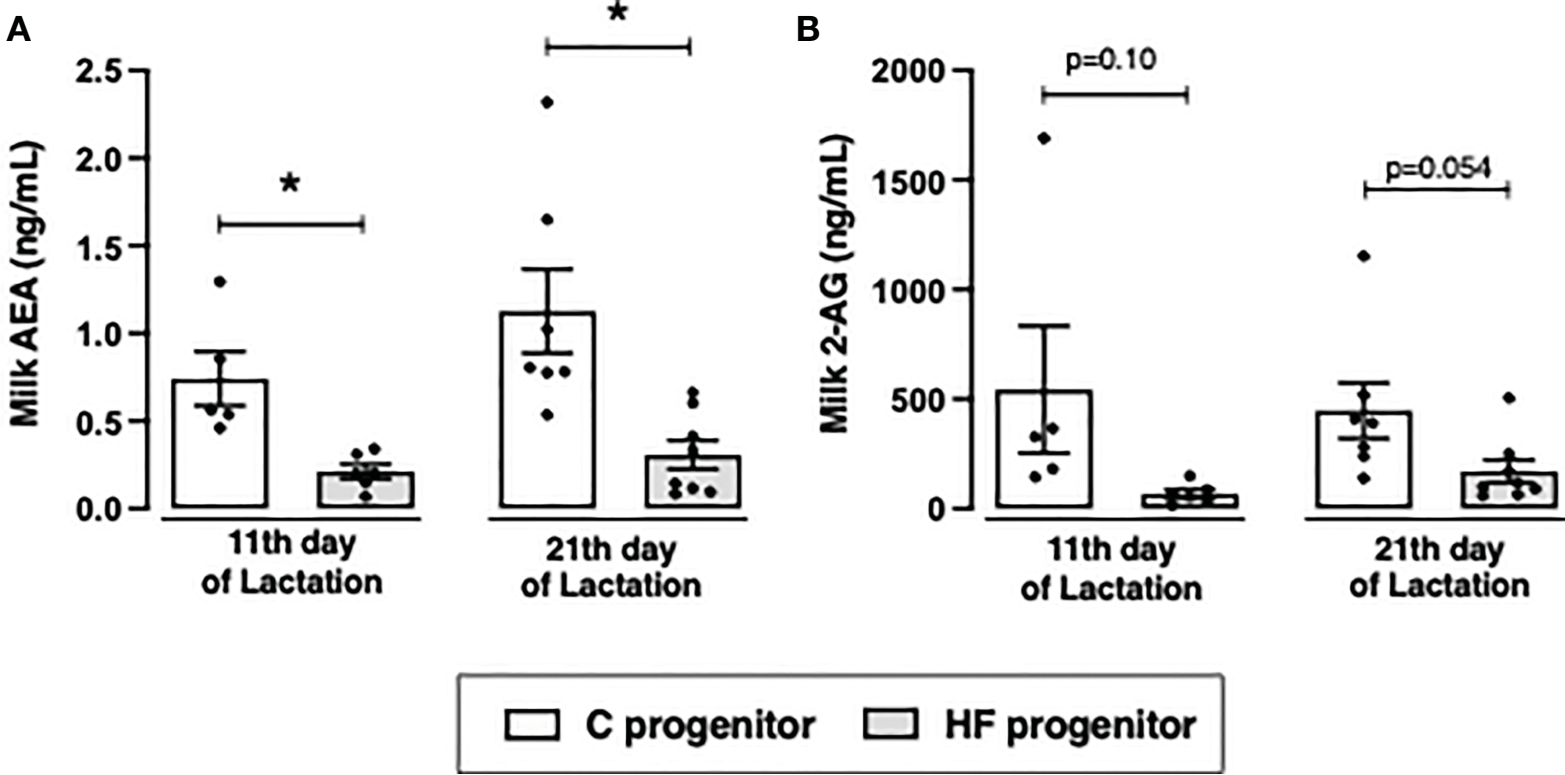
Effect of high-fat (HF) diet on the content of endocannabinoids in the milk of rat progenitors. **(A)** Milk anandamide (AEA) and **(B)** 2-arachidonoylglycerol (2-AG) in the postnatal days 11 and 21 of control (C) and HF progenitors. Data are presented as mean ± standard deviation and statistically significant differences were determined by unpaired Student’s *t* test. *p < 0.05.

Maternal HF diet decreased AEA content in the milk at postnatal day 11 (- 71%, *p<0.05*) and 21 (- 72%, *p<0.05*) compared to maternal C diet (**Figure 4A**). Maternal HF diet also decreased milk 2-AG content at postnatal day 11 (- 87%, *p = 0.10*) and 21 (- 62%, *p = 0.054*) (**Figure 4B**).

**Figure 5:**
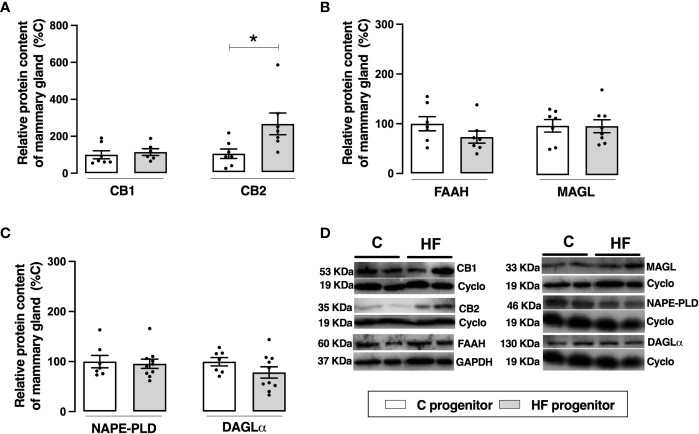
Effect of high-fat (HF) diet on the endocannabinoid system in the mammary gland of rat progenitors. **(A)** Type-1 cannabinoid receptor (CB1) and type-2 cannabinoid receptor (CB2) protein content; **(B)** fatty acid amide hydrolase (FAAH) and monoacylglycerol lipase (MAGL) protein content; **(C)** N-acyl phosphatidylethanolamine phospholipase D (NAPE-PLD) and diacylglycerol lipase (DAGL) protein content of mammary gland of control (C) and HF progenitors. **(D)** representative blots are shown. Data are expressed as percentage of control group (set at 100%) ± standard deviation. Statistically significant differences were determined by unpaired Student’s *t* test. *p < 0.05.

Maternal HF diet did not change the content of CB1 but increased the content of CB2 in the mammary tissue (+ 1.6-fold %, *p<0.05*). Maternal HF diet did not change the endocannabinoid degrading enzymes FAAH or MAGL neither the synthesizing enzymes NAPE-PLD or DAGL (**Figure 5**).

The analysis of fatty acid profile in the milk showed that maternal HF diet increased the content of saturated fatty acid in late lactation (postnatal day 21) (2-3-fold increase, *p<0.05*) (**Table 4**). In addition, maternal HF diet decreased the milk content of the polyunsaturated fatty acids (PUFA) eicosapentanoic acid (n3, -30%, *p<0.05*), linoleic acid (n6, -51%, *p<0.05*), arachidonic acid (n6, -40%, *p<0.05*), docosatetraenoic acid (n6, -55%, *p<0.05*) in mid-lactation (postnatal day 11) (**Table 4**).

**Table 4:** Fatty acid profile in breast milk of lactating rats at mid-lactation (11^st^ day) and late lactation (21^st^ day).

		11st day			21st day		
		C	HF	p	C	HF	p
Fatty acid profile (μg/μL)	C12:0	13.70 ± 1.02	8.643 ± 1.32*	0.04	10.28 ± 2.72	8.703 ± 1.45	0.65
	C15:0	0.487 ± 0.03	1.061 ± 0.26	0.10	0.490 ± 0.13	1.398 ± 0.36*	0.04
	C16:0	27.57 ± 3.0	24.19 ± 3.03	0.47	14.64 ± 2.75	27.40 ± 3.48*	0.02
	C17:0	0.323 ± 0.02	0.557 ± 0.11	0.13	0.292 ± 0.07	0.653 ± 0.10*	0.02
	C18:0	4.657 ± 0.52	6.784 ± 0.89	0.11	3.164 ± 0.52	8.017 ± 0.88*	0.01
	C16:1	2.071 ± 0.51	3.615 ± 0.7	0.15	2.093 ± 0.60	5.413 ± 1.39	0.06
	C18:1	19.64 ± 2.85	22.50 ± 3.32	0.55	13.33 ± 2.50	29.46 ± 3.40*	0.01
	C20:5 n3	0.279 ± 0.02	0.153 ± 0.02*	0.02	0.108 ± 0.01	0.138 ± 0.01	0.16
	C22:5 n3	0.301 ± 0.03	0.149 ± 0.03	0.04	0.132 ± 0.02	0.143 ± 0.02	0.78
	C22:6 n3	0.212 ± 0.02	0.136 ± 0.03	0.16	0.163 ± 0.04	0.125 ± 0.02	0.18
	C18:2 n6	27.65 ± 1.69	13.54 ± 1.99*	0.01	21.10 ± 3.99	18.17 ± 0.02	0.56
	C20:4 n6	1.951 ± 0.07	1.171 ± 0.13*	0.01	1.466 ± 0.29	1.156 ± 0.14	0.42
	C22:4 n6	0.551 ± 0.07	0.250 ± 0.06*	0.04	0.277 ± 0.06	0.241 ± 0.04	0.69
	ARA/EPA+DHA	4.038 ± 0.56	4.077 ± 0.40	0.93	5.438 ± 1.27	4.414 ± 0.578	0.18

Data presented as mean ± standard deviation. Statistically significant differences were determined by unpaired test t (C vs HF), considering *p < 0.05. n=3-5 samples per group. Lauric acid (C12:0), pentadecanoic acid (C15:0), palmitic acid (C16:0), heptadecanoic acid (C17:0), stearic acid (C18:0), palmitoleic acid (C16:1), oleic acid (C18:1), eicosapentanoic acid (EPA, C20:5 n3), docosapentaenoic acid (C22:5 n3), docosahexaenoic acid (DHA, C22:6 n3), linoleic acid (C18:2 n6), arachidonic acid (ARA, C20:4 n6), docosatetraenoic acid (C22:4 n6). bold values highlight the statistical significance.

### 3.4 ECS and dopamine signaling in the NAc of weanling offspring

**Figure 6:**
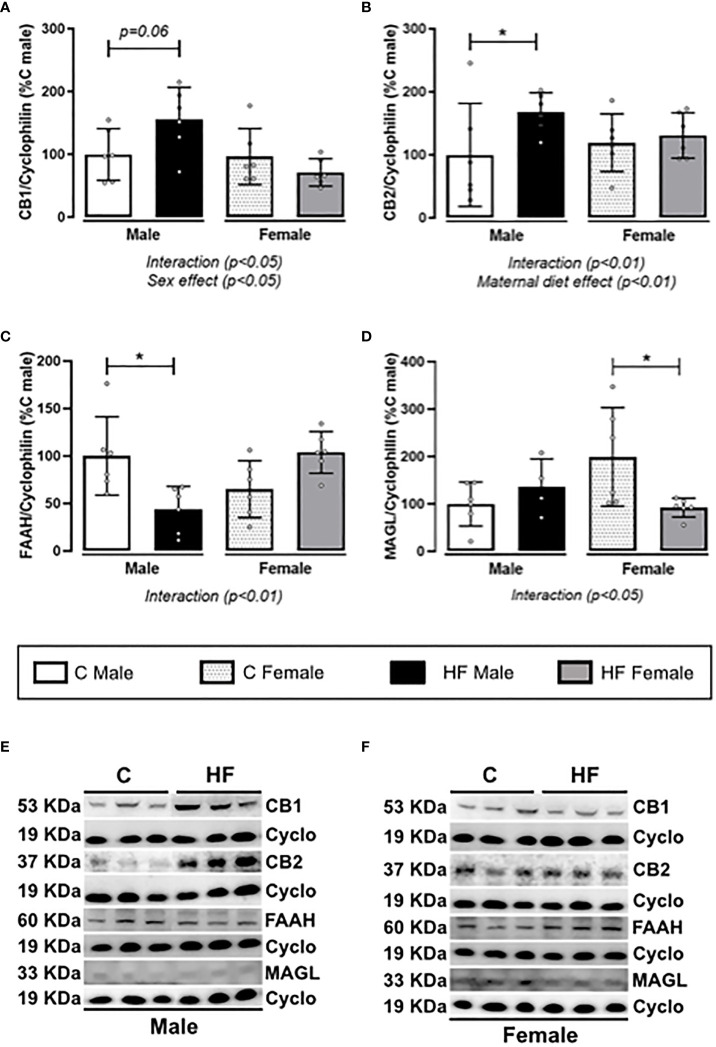
Effect of maternal high-fat (HF) diet on the endocannabinoid signaling in the Nucleus Accumbens (NAc) of weanling offspring. **(A)** Type-1 cannabinoid receptor (CB1); **(B)** type-2 cannabinoid receptor (CB2); **(C)** fatty acid amide hydrolase (FAAH); and **(D)** monoacylglycerol lipase (MAGL) protein content in the NAc of control **(C)** and HF male and female offspring at weaning. **(E, F)** representative blots are shown. Data are expressed as percentage of control male group (set at 100%) ± standard deviation. Statistically significant differences were determined by two-way ANOVA followed by Bonferroni’s test. *p < 0.05.

Maternal HF diet differently altered the ECS in the NAc of male and female weanling offspring, represented for interaction effect observed in all parameters analyzed (*p<0.05*). In addition, there is a marked sex effect on the CB1 content (*p<0.05*). In male offspring, maternal HF diet increased CB1 (+ 56%, *p = 0.06*) and CB2 (+ 67.6%, *p<0.05*), while maternal HF diet did not change cannabinoid receptors in female offspring (**Figures 6A, B**). Maternal HF diet decreased FAAH content (- 56.1%, *p<0.05*) (**Figure 6C**) in the NAc of males with no changes in MAGL (**Figure 6D**), compared to sex-matched controls. In female HF offspring, we observed an different profile, with decreased MAGL (- 50%, *p<0.05*) (**Figure 6D**) with no changes in FAAH content (**Figure 6C**), compared to sex-matched controls.

**Figure 7:**
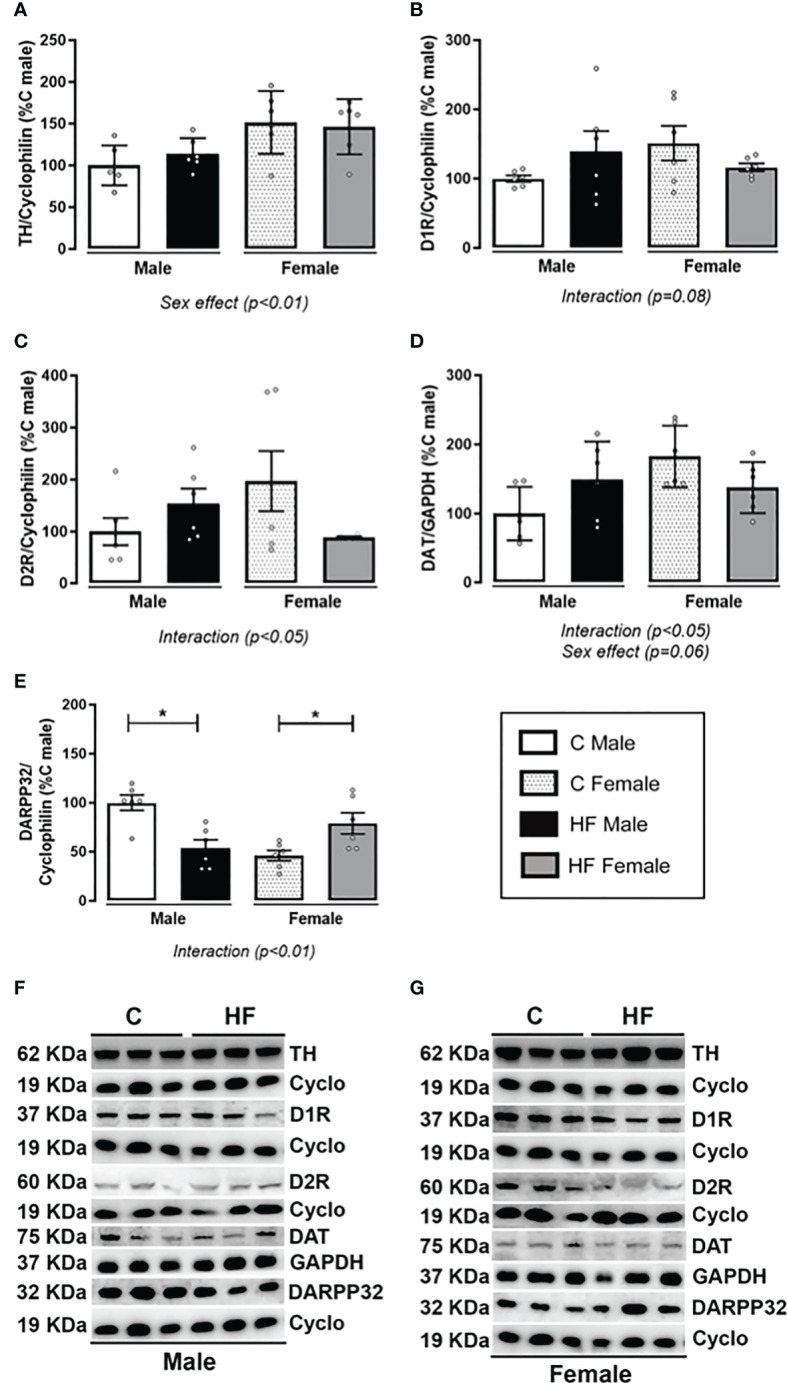
Effect of maternal high-fat (HF) diet on the dopamine signaling in the Nucleus Accumbens (NAc) of weanling offspring. **(A)** Tyrosine hydroxylase (TH); **(B)** type-1 dopamine receptor (D1R); **(C)** type-2 dopamine receptor (D2R); **(D)** dopamine transporter (DAT); and **(E)** cAMP-regulated neuronal phosphoprotein-32 kDa (DARPP-32) protein content in the NAc of control **(C)** and HF male and female offspring at weaning. **(F)** and **(G)** show the representative blots. Data are expressed as percentage of control male group (set at 100%) ± standard deviation. Statistically significant differences were determined by two-way ANOVA followed by Bonferroni’s test. *p < 0.05.

Maternal HF diet differently altered the dopamine signaling in the NAc depending on offspring sex, since there was an interaction effect (*p<0.05*) in the content of dopamine receptors (D1R and D2R), dopamine transporter (DAT) and cAMP-regulated neuronal phosphoprotein-32 kDa (DARPP-32) (**Figures 7B-E**, respectively). In these parameters, the male HF offspring presented a profile of increase while female HF offspring presented profile of decrease. There was a sex effect on the tyrosine hydroxylase (TH) content, a limiting enzyme of the dopamine synthesis, with female offspring presenting increased content (Sex effect, *p<0.01*), regardless of maternal diet (**Figure 7A**). Similarly, female HF offspring have increased DAT content (+ 82.5%, *p<0.05*) compared to male offspring, regardless of maternal diet (**Figure 7D**). Maternal HF diet decreased DARPP32 content in male offspring (- 46.3%, *p<0.05*) while increased in female offspring (+ 71.2%, *p<0.05*) (**Figure 7E**).

### 3.5 Food preference test

**Figure 8:**
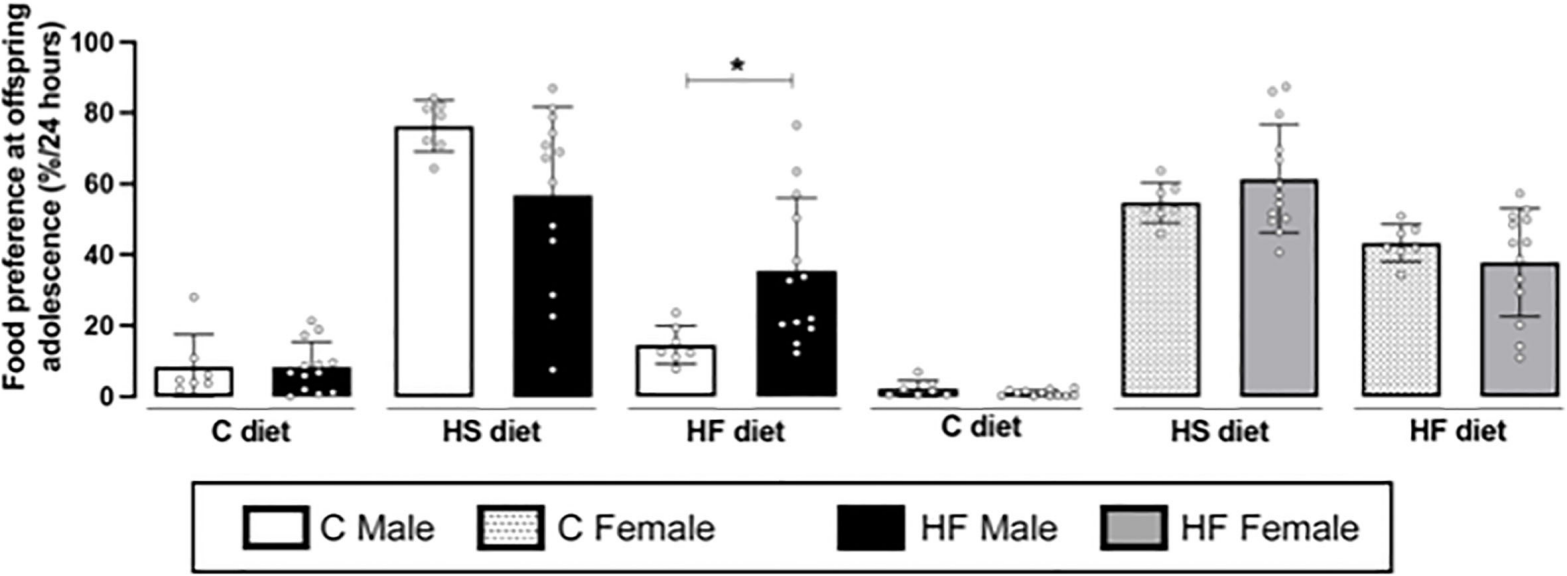
Effect of maternal high-fat (HF) diet on the food preference of rat offspring at adolescence. Intake of control **(C)**, high sucrose (HS) and HF diets of male and female offspring born from progenitors receiving control **(C)** or HF diet. Statistically significant differences were determined by unpaired Student’s *t* test. *p < 0.05.

Maternal HF diet induced higher preference for HF diet (+ 2.4-fold, *p<0.05*) in adolescent male offspring, while did not change the preference for C or HS diets (**Figure 8**). In the female offspring, maternal HF diet did not alter food preference in the adolescence (**Figure 8**) Representative blots are show in **Figure 6E, F**.

## 4 Discussion

In the present study, we characterized the presence of lipid endocannabinoids in breast milk of lactating rats and how maternal HF diet changes the levels of milk AEA and 2-AG in different time points of lactation. We also showed that maternal HF diet modulates the cannabinoid and dopamine signaling in the NAc of weanling rat offspring in a sex-dependent manner, which may be associated with the increased preference for dietary fat observed in male but not in female adolescent rats Representative blots are show in **Figure 7F** and **Figure 6G**.

The HF diet increased body weight gain of female progenitors prior mating but did not change weight gain during gestation or lactation. HF diet also did not change serum metabolic parameters (glycemia, triglycerides and cholesterol) in the dams at weaning. These data corroborate our previous results showing increased total body fat but not body weight of the dams before mating (23). In contrast, the literature mainly shows that HF diet increases body weight, adiposity, glycemia, dyslipidemia, insulinemia and leptinemia in female rats prior pregnancy, and several serum alterations last until weaning (46–49). An important characteristic of the present study is that the HF diet used is isocaloric in comparison to the control diet (~3.9 kcal/g), while most of the studies published in the literature are performed using a high-fat and hypercaloric diet (over 5 kcal/g). Additionally, the diets used in several other studies are also often enriched in simple sugar (fructose or sucrose). This study was performed using a mild HF diet (29% fat), which did not induce a severe metabolic phenotype in the dams, but it was able to induce a marked phenotype in the offspring. On the other hand, we have demonstrated that maternal HF diet increases the content of macronutrients (lipids, protein, lactose) in the breast milk across lactation, which seems to be an important imprinting factor for the offspring (23).

During lactation, male and female offspring developed early obesity characterized by increase of body weight and adiposity at weaning in parallel to increased glycemia and insulin levels. This phenotype has also been shown by our previous studies (23, 34) and by several other research groups (47, 50–52). In the present study, maternal HF diet decreased birth weight only in female offspring. When evaluating the growth trajectories of both sexes, it is evident that female offspring present a more accelerated weight gain during lactation. It is possible that maternal HF diet differentially alters placenta function according to the offspring sex, resulting in different programming models. We speculate that male HF offspring is mainly exposed to changes in milk, while female HF offspring already present significant alterations before milk changes exposure. This difference in growth rates may contribute to the higher magnitude of adipose tissue accumulation observed in female HF offspring at weaning, compared to HF males. In addition, female HF offspring remain heavier compared with the control offspring during the adolescence, but this phenotype was not observed in male offspring. Interestingly, in a previous paper, we have demonstrated that maternal HF diet has a more pronounced effect on adipose tissue of adult female offspring compared with male offspring. In parallel, adult HF female have increased expression/content of CB1 in visceral and subcutaneous adipose tissue at postnatal day 180, associated with increased estrogen receptor binding to the Cnr1 gene (35). Despite presenting greater adiposity, adult female HF offspring do not have higher levels of triglycerides, glycemia or liver steatosis (36), suggesting that this energy accumulation (mainly in subcutaneous adipose tissue) is relatively controlled in terms of systemic metabolic complications. Differently, adult HF males present such alterations (36).

Surprisingly, in the animal set used for this study, we did not observe increased leptin levels as expected by the higher adiposity of the HF offspring and our previous results (23, 34). We speculate that this variance among the experimental sets might be related to the magnitude of increase in adiposity. In the present experiment, HF offspring presented an increment of 2-3-fold in adiposity, while in the previous sets it reached 4-fold increase.

Lactation is a critical window for metabolic programming in rodents, and alterations in the milk macronutrients and hormones contribute to detrimental phenotypes (50, 51, 53). In rodents, the completion of hepatocyte differentiation and bile duct formation as well as nephrogenesis take place during lactation (54). The peak of adipose tissue development also occurs during lactation, when the number and the size of adipocytes increase gradually. After puberty, the adipocyte number and size remain relatively stable (55). Similarly, during lactation occurs the peak of brain development, which reaches 90% of adult weight around weaning, when there is a peak in synaptic density and myelination rate (54).

In rodents, maternal HF diet only during lactation recapitulates the offspring metabolic phenotype programmed by HF diet throughout gestation and lactation (47, 56), highlighting the key contribution of lactation. In humans, the exclusive breastfeeding for the first six months of life prevents morbidity and mortality as well as promotes the physical and mental health of infants (57). Maternal obesity alters the milk fatty acid profile (increased saturated fatty acids and decreased n3 PUFA) associated with infant cognitive impairment (25).

In the present study, maternal HF diet decreased the milk content of n3 and n6 PUFA at mid-lactation (postnatal day 11). Castillo et al. also demonstrated that maternal HF (40% fat) decreases n3 PUFA but does not change the AA (n6 PUFA) levels in rat breast milk at mid-lactation (postnatal day 10) (58). In a similar rat model, maternal obesity (25% fat) decreases n3 PUFA (EPA and DHA) milk content while increases milk AA at late lactation (51). Breast milk PUFA are crucial for postnatal growth and neurodevelopment, and about half of the dry weight of the brain is made up by lipids, of which 20–25% are PUFA (59, 60). The milk composition of fatty acids is a combination of dietary lipids and the *de novo* fatty acid synthesis in the mammary gland or in other maternal tissues, such as liver (51, 61–63). It was demonstrated that the mammary gland deletion of fatty acid synthase (FAS) induces the premature involution of lactating mammary gland and decreases medium- and long-chain fatty acids and total fatty acid content in breast milk of mice (62). In the present model, we did not observe changes in lipogenic enzymes in the mammary gland (data not shown), suggesting a major contribution of the diet to the milk fatty acid profile. However, Bautista et al. showed that maternal obesity decreases the content of total n3 and n6 PUFA in the mammary gland associated with decreased expression of the enzyme delta 5 desaturase at weaning (51), and Castillo et al. observed that maternal HF diet increases the mRNA expression of FAS and lipoprotein lipase (lipogenic factors) in the mammary gland (58).

The imbalance between n6 and n3 PUFA can also affect the levels of endocannabinoids, since AEA and 2-AG are derived from membrane phospholipids containing AA (7). We observed that maternal HF diet parallelly reduced AA and the endocannabinoid levels in the breast milk, and this association was more robust in mid-lactation. To investigate possible causes of the decreased milk endocannabinoid levels, we analyzed the ECS expression in the mammary gland. However, maternal HF did not affect the content of the synthesizing enzymes (NAPE-PLD and DAGLa) or the degrading enzymes (FAAH and MAGL). Thus, other metabolic factors in the dams, including the uptake and transport of endocannabinoids in the mammary gland, may be involved in this profile. Maternal HF diet increased the CB2 content in the mammary gland, possibly as an adaptation to counteract local inflammation (64) induced by HF diet.

There are very few studies in the literature showing the presence of endocannabinoids in human milk (28–30, 65–67), and to the best of our knowledge, this is the first study evaluating these bioactive lipids in rat milk. However, the physiological significance of the presence of lipid endocannabinoids in breast milk remains to be elucidated. In a cohort from Guatemala, the levels of PEA in mature milk increases across lactation and there is a positive correlation between milk AA and arachidonoyl glycerol (AG) (67), corroborating our data. On the other hand, in a small human cohort, it was demonstrated that maternal obesity or overweight do not alter the 2-AG levels in mature milk, and higher milk levels of 2-AG are observed during daylight compared to night levels (29). In addition, milk levels of OEA and PEA are lower in the mothers of four-month babies with higher body weight compared to the ones presenting lower body weight (66). This data suggests a negative correlation between milk endocannabinoids and body weight in lactation, like the profile observed in the present study.

We found that concentration of AEA and 2-AG in rat milk was comparable to that observed in human milk (28, 29) but slightly higher, possibly because rat milk has higher fat content compared to human milk (51, 68–70). Notedly, the relative amount of 2-AG in rat milk was found 1,000-fold more concentrated than AEA. This profile has been demonstrated in other biological samples, such as brain (71, 72). We speculate that decreased levels of endocannabinoids in breast milk may result in decreased exposure to these lipids in the developing brain impairing maturation like what is known for other milk components such as leptin (73, 74). Additionally, maternal exposure to *Cannabis* during pregnancy and lactation is related to the presence of phytocannabinoids in umbilical cord blood and breast milk and are associated with impaired offspring social, behavioral, and cognitive development (75).

Maternal HF diet increased the content of saturated fatty acids in the milk at late lactation, which can be a direct effect of the maternal diet rich in lard (76). The high exposure to saturated fatty acids has been related to insulin resistance (77), and we observed hyperglycemia and hyperinsulinemia in the HF offspring at weaning, suggesting the contribution of the breast milk. We have also demonstrated that maternal HF diet induces hypothalamic leptin resistance in the offspring at weaning (23, 33) and adulthood (39). High-fat diets rich in long-chain saturated fatty acids, mainly C16:0, contribute to hypothalamic inflammation, which has been shown to be causative of central leptin resistance, contributing to obesity development (78, 79). Leptin signaling impairment is associated with increased central cannabinoid signaling (80), and we have also demonstrated that maternal HF diet increases CB1 content in the hypothalamus of male newborn rats (32).

Besides the evident importance of the hypothalamus on homeostatic feeding and energy expenditure regulation, the LH-VTA-NAc pathway regulates dopaminergic signaling and the hedonic aspects of food intake and reward (81). The LH-VTA-NAc pathway is highly regulated by GABA and glutamate neurons expressing CB1 (80, 82). Dopaminergic neurons in the VTA project to the NAc, where they release dopamine to bind to D1R and D2R and promote reward. The activity of dopamine neurons is regulated by GABAergic inputs projecting from the NAc and orexin-A neurons from the LH. Dopamine neurons synthesize 2-AG, which binds to CB1 expressed presynaptically in GABAergic and glutamatergic neurons, fine tuning dopamine neuron activity. In addition, the activity of GABAergic neurons projecting from the NAc to VAT is directly regulated by glutamatergic neurons expressing CB1. Therefore, increased CB1 signaling in the LHA-VTA-NAc circuitry is associated with increased dopamine release and reward (7).

In the present study, maternal HF diet increased cannabinoid receptors in the NAc of male weanling rats but not in females. In addition, it was observed a parallel decrease of the degrading enzymes FAAH and MAGL in males and females, respectively, suggesting an increase of tissue levels of endocannabinoids. Increased cannabinoid signaling in the NAc can result in inhibition of GABA inputs to the dopamine neurons in the VAT, thus increasing dopamine release as well as palatable food-seeking behavior (17). We also analyzed the dopamine signaling in the NAc of the weanling offspring. Maternal HF diet induced a sex-specific regulation of this signaling, characterized by the “interaction” observed in the two-way ANOVA analysis, with increased content of dopamine receptors and DAT in males but not in females. However, maternal HF diet decreased the content of the DARPP-32 in males and increased in females. Because DARPP-32 signals the downstream pathway of both D1R and D2R, this result suggests that the intracellular signaling is impaired in male HF offspring. In chronic models of diet-induced obesity, it has been demonstrated a hyporesponsiveness of the dopaminergic mesolimbic circuitry (83, 84) associated with higher vulnerability to preference for highly palatable diets and compulsive behavior (84, 85). We speculate that decreased DARPP-32 may be an initial response to maternal HF diet that could contribute to increased appetite for palatable food, which would probably be followed by a downregulation of the dopamine receptors in the long-term.

Here, changes in the cross-talk between cannabinoid and dopamine signaling were associated with increased preference for dietary fat in the HF male offspring at adolescence, a critical window for the developmental origins of health and disease (DOHaD) (86), neurodevelopment and addiction susceptibility (9, 87). Previously, we have demonstrated that adult male and female HF offspring present higher preference for fat (32), and the present data showed that this phenotype is already evident during adolescence in males. Interestingly, male HF offspring develop a more pronounced metabolic phenotype compared to females, presenting liver steatosis, insulin resistance and dyslipidemia at adulthood (36). In this model, nutritional intervention (fish oil) during adolescence represents an important approach to prevent part of the metabolic dysfunctions (76), highlighting this critical window.

Other studies have demonstrated that maternal HF diet alters the dopaminergic system in limbic areas of offspring contributing to preference to highly palatable foods (83, 88–94). In contrast, adolescent and adult male offspring from dams exposed to hypercaloric-hypoproteic diet present decreased chocolate preference associated with decreased endocannabinoid levels in the hypothalamus (95). In a programming model of early weaning (early undernutrition), it was demonstrated that adult male offspring present increased “voracity” for high-fat diet (30 minutes of free choice diet) but increased preference for sugar (12h of free choice diet) while no effect was observed in females (96). In parallel, early weaning induced decreased D2R in the NAc of adult male without marked changes in the cannabinoid signaling in the hypothalamus, but the ECS in the NAc was not evaluated (96).

In the present study, differently of adolescent males, female HF offspring had no alterations in the food preference, however, they showed decreased MAGL, D2R and DAT, and increased DARPP-32 content in the NAc at weaning, suggesting higher endocannabinoids and dopamine signaling. These data demonstrate sex-specific effects of maternal HF diet on the offspring. In addition, the two-way ANOVA analysis showed a “sex effect” on the CB1, TH and DAT content along with interaction between “maternal diet” and “offspring sex” in almost all ECS and dopamine signaling markers. There are several mechanisms that could potentially contribute to the sex differences observed in the ECS and dopamine signaling. Previously, it was demonstrated that sex hormones participate of brain development during the intrauterine life, and testosterone and estrogen surges are observed in neonate boys and girls, respectively, representing a “mini-puberty” (97). In our animal model, regardless of changes in the sex hormone circulating levels, we have demonstrated that maternal HF diet increases the acetylation levels of the cannabinoid receptor 1 gene (*Cnr1*) promoter associated with increased binding of the androgen receptor (AR) and *Cnr1* mRNA levels only in the hypothalamus of male offspring (33). A similar mechanism may be involved in the sex-specific changes observed for the NAc in the present study. Considering the dopamine signaling, the estradiol receptor β (ERβ) is co-localized with TH in VAT neurons of males and females, which could contribute to higher TH expression in female (98). Estradiol receptors (ERα/β) are also present in neuronal membranes, where they interact with metabotropic glutamate receptor to decrease the inhibitory GABAergic input from NAc to VTA with consequent increase of dopamine release in females (99). However, more studies are needed to explore the direct regulation of sex hormones on ECS and dopamine signaling, possibly using *in vitro* models.

This study is not without limitations. It was not possible to identify the cause of decreased levels of AEA and 2-AG in rat milk induced by HF diet, and further experiments are needed (for example testing ECS enzyme activity). Also, it would be important to quantify the endocannabinoid levels in that NAc, which was not possible at this experimental design due to the limited amount of tissue obtained with the “punch” technique. Lastly, as mentioned, the investigation of molecular mechanisms to explain the sex differences in deeper is desired. On the other hand, this study brings novelty to the field of the cannabinoid research and DOHaD since it characterized the presence of endocannabinoids in breast milk of rats and its possible causative role on the development of food preference.

## 5 Conclusion

Contrary to our hypothesis, maternal HF diet reduced AEA and 2-AG in rat breast milk. We speculate that decreased endocannabinoid levels in early development may alter neuronal maturation in the offspring. Maternal HF diet induced adaptive sex-specific changes in the NAc of the weanling rats, with males presenting increased cannabinoid receptors, possibly in response to the decreased milk endocannabinoid levels, and females presenting increased dopamine intracellular signaling. However, these molecular changes were only associated with increased preference for fat in the adolescent male offspring, but it is probably involved in the adult preference for fat in the females previously shown. Because the ECS signaling stimulates appetite for palatable foods and reward as well as adiposity and lipogenesis, the increase of this pathway at weaning may contribute to the earlier metabolic phenotype in males, which is known to present a more severe dysmetabolism in the long-term.

## Data availability statement

The original contributions presented in the study are included in the article/supplementary material. Further inquiries can be directed to the corresponding author.

## Ethics statement

The animal study was reviewed and approved by Animal Care and Use Committee of the Carlos Chagas Filho Biophysics Institute (process number 059/19) of the Federal University of Rio de Janeiro.

## Author contributions

CD-R, JC, YO and LF: Conceptualization, Methodology, Formal analysis, Investigation, and Writing Original Draft. GA: Methodology – Fatty Acid Profile. JW: Methodology – Experimental model design and handling. GS and HP: Methodology – Endocannabinoid quantification. CP-M: Conceptualization, Resources, Writing - Review and Editing. MA: Conceptualization, Supervision, and Writing - Review and Editing. IT: Conceptualization, Resources, Supervision, Project administration, Funding acquisition, and Writing - Review and Editing. All authors contributed to the article and approved the submitted version.
